# The role of magnetoencephalography in preoperative localization and postoperative outcome prediction in patients with posterior cortical epilepsy

**DOI:** 10.1111/cns.14602

**Published:** 2024-02-08

**Authors:** Guiliang Hao, Hao Yan, Xueyuan Wang, Runshi Gao, Yansong Xue, Xiating Zhang, Duanyu Ni, Wei Shu, Liang Qiao, Liu He, Tao Yu

**Affiliations:** ^1^ Department of Functional Neurosurgery, Beijing Institute of Functional Neurosurgery, Xuanwu Hospital Capital Medical University Beijing China; ^2^ Department of Neurology, Xuanwu Hospital Capital Medical University Beijing China

**Keywords:** magnetoencephalography, posterior cortex epilepsy, presurgical localization, surgical outcome

## Abstract

**Objective:**

We aimed to explore the value of magnetoencephalography in the presurgical evaluation of patients with posterior cortex epilepsy.

**Methods:**

A total of 39 patients with posterior cortex epilepsy (PCE) and intact magnetoencephalography (MEG) images were reviewed from August 2019 to July 2022. MEG dipole clusters were classified into single clusters, multiple clusters, and scatter dipoles based on tightness criteria. The association of the surgical outcome with MEG dipole classifications was evaluated using Fisher's exact tests.

**Results:**

Among the 39 cases, there were 24 cases of single clusters (61.5%), nine cases of multiple clusters (23.1%), and six cases of scattered dipoles (15.4%). Patients with single dipole clusters were more likely to become seizure‐free. Among single dipole cluster cases (*n* = 24), complete MEG dipole resection yielded a more favorable surgical outcome than incomplete resection (83.3% vs. 16.7%, *p* = 0.007). Patients with concordant MRI and MEG findings achieved a significantly more favorable surgical outcome than discordant patients (66.7% vs. 33.3%, *p* = 0.044), especially in single dipole cluster patients (87.5% vs. 25.0%, *p* = 0.005).

**Significance:**

MEG can provide additional valuable information regarding surgical candidate selection, epileptogenic zone localization, electrode implantation schedule, and final surgical planning in patients with posterior cortex epilepsy.

## Graphical abstract

Different MEG dipole clusters have varying implications for the surgical outcome of patients with posterior cortical epilepsy. When MEG showed a single cluster, the likelihood of being postoperatively seizure‐free was significantly increased.

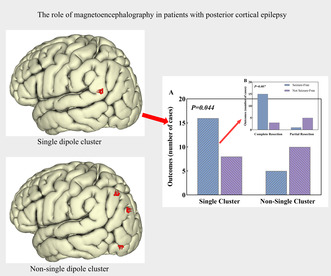

## 1 INTRODUCTION

The term “posterior cortex epilepsy” (PCE) refers to epilepsy originating from the occipital, parietal, or posterior part of the temporal lobe, or from any combination of these regions.^1^, ^2^, ^3^ Because of the lack of clear anatomical or neurophysiological distinctions among these cortical areas, the epileptogenic regions may not always be confined strictly within the anatomical boundaries of the occipital, parietal, or posterior temporal lobe.^1^, ^4^ Therefore, identifying the precise localization of the epileptogenic area remains a challenge, thereby leading to a relatively lower incidence of surgical treatment for epilepsy arising from the posterior cortex, as compared to those originating from the anterior temporal and frontal regions.^5^ In addition, it is noteworthy that epilepsy surgery for PCE has been associated with less favorable postoperative outcomes compared to temporal lobe epilepsy. Long‐term favorable outcomes following PCE surgery have been reported to vary from 17% to 74%.^1^, ^3^, ^6^, ^7^, ^8^, ^9^

Electrophysiology, particularly techniques such as electroencephalography (EEG), is currently the primary method used to localize the epileptogenic focus during preoperative evaluations. This method exhibits distinct sensitivities based on the spatial organization and functional aspects of the generator, as demonstrated for both physiologic activities^10^ and interictal spikes.^11^ However, surface EEG offers limited localizing information in PCE and is often deemed misleading.^2^, ^12^ In some patients, the combination of EEG and imaging, as well as symptomatology, still presents difficulties in locating the epileptic zone. In this circumstance, an effective solution is to employ stereo‐electroencephalography (SEEG), which can directly record cortical activity and localize interictal spikes. However, because SEEG techniques have limited spatial sampling, covering <5% of the whole brain,^13^, ^14^, ^15^ the effectiveness of SEEG depends on the quality of preoperative evaluations in formulating the electrode‐implantation plan.

Consequently, there is a need for further exploration and refinement of methods aimed at localizing the epileptogenic area in PCE patients. Magnetoencephalography (MEG) serves as a non‐invasive clinical tool that has been reported to provide additional, and sometimes unique, information in localizing the epileptogenic zone (EZ).^16^, ^17^, ^18^, ^19^, ^20^, ^21^ Compared to EEG, MEG exhibits better spatial resolution due to the ability of magnetic signals to traverse the skull, skin, and other tissues without experiencing distortion.^22^ In contrast, electrical signals detected by EEG are subject to distortion when passing through the skull and other tissues. Another notable distinction between MEG and EEG lies in their respective sensitivities to brain activity. MEG exhibits heightened sensitivity towards neural activity occurring within the sulci, or grooves, of the brain, whereas EEG demonstrates greater sensitivity towards activity taking place on the brain's surface.^23^, ^24^, ^25^ Besides, simulated computation analysis suggests that MEG can record 95% of cortical activity, a significantly higher proportion than EEG, which is more attuned to radial sources.^26^ Despite the potential of MEG, few data are available regarding the role of MEG in the presurgical evaluation of patients with PCE.

In this study, our objective was to explore the additional value of MEG in the presurgical localization of patients with PCE.

## 2 METHODS

### 2.1 Patients

We retrospectively reviewed the data of patients who received surgical treatment for epilepsy at the Beijing Institute of Functional Neurosurgery (Beijing, China) between August 2018 and July 2022. Patients who met the following criteria were selected: (1) refractory patients who underwent a formal presurgical evaluation for epilepsy surgery and with complete clinical data (including but not limited to preoperative MRI, MEG, and EEG); (2) patients who underwent surgical resection involving the posterior cortex and with complete postoperative CT or MRI data; and (3) patients with a follow‐up period of 12–48 months. Among the 854 patients reviewed, 39 (4.6%) satisfied the inclusion criteria. All patients provided written informed consent, and legal guardians provided consent for underage subjects.

### 2.2 Presurgical evaluation

#### 2.2.1 Neuroimage

All patients underwent high‐resolution MRI in a 3.0T MR scanner. Spin‐echo T1‐weighted, T2‐weighted, and fluid‐attenuated inversion recovery sequences, and three‐dimensional anatomic T1‐weighted axial, sagittal, and coronal sequences covering the whole brain volume with a 1‐mm section thickness were collected. Positron emission tomography–computed tomography (PET–CT) was performed to localize the epileptic zone in most patients (*n* = 30).

#### 2.2.2 EEG

Long‐term scalp video electroencephalography (v‐EEG) monitoring with electrodes placed according to the international 10–20 system was routinely performed. Usually, at least three habitual seizures were recorded for patients during the long‐term monitoring.

#### 2.2.3 MEG

All patients underwent MEG using a 306‐channel whole‐head system (Neuromag Helsinki, Finland), and simultaneous EEGs were recorded for 60 min. The sampling frequency of MEG was 1000 Hz. Before data acquisition commenced, three coils were attached to the bilateral preauricular points and nasion of each subject. Then patients were required to lie comfortably in a supine position with their eyes closed during the MEG recordings. Then, 3D MRI with three fiduciary marks in the same positions during MEG recordings was obtained. The MEG signal was analyzed by the single equivalent current dipole (SECD) method using Neuroimage software (Elekta, Stockholm, Sweden). The conventional spike discharges were visually identified as waveforms, with a band‐pass filter of 3–70 Hz. The MEG results were co‐registered with the patient's MRI to visualize epileptic foci. Sources of spikes with goodness‐of‐fit values >85% were considered significant.^27^

Dipole clusters were categorized into three types depending on their “tightness” information. In this study, a cluster was defined as at least five dipoles within a 1 cm^3^ region, as used in a prior study.^16^, ^28^ A single cluster was defined as five or more dipoles located within a single gyrus or two adjacent gyri.^29^ Multiple clusters were defined by more than one cluster located in different and not adjacent gyri. Scattered dipoles were defined by less than four dipoles located in different gyri. The open source software and toolboxes Freesurfer,^30^ Desikan‐Killiany (DK) Atlas,^31^ and 3D slicer^32^ were used for image processing and visualization. The gyri were defined using the Desikan‐Killiany (DK) atlas with the help of Freesufer.

All the clinical information, neuroimaging data, v‐EEG data as well as MEG findings were analyzed by a special group to localize the epileptogenic zones (EZs). If necessary, this group will make the plan for further SEEG implanting for patients according to their localizing hypothesis.

### 2.3 Intracranial recording

In this study, 13 patients received stereotactic implantation and SEEG recording, with the number of recording contacts ranging from 8 to 20 for each electrode (contact length: 2 mm, contact spacing: 1.5 mm). Using open‐source software and toolboxes SPM12,^33^ Freesurfer,^30^ and 3D slicer,^32^ preoperative high‐resolution MRI images were registered with postoperative high‐resolution CT images. The electrode contacts were reconstructed using the CT images. The intracranial EEG sampling rate was set at 1024 Hz. The duration of video‐EEG monitoring ranged from 3 to 20 days, and at least three habitual seizures were recorded for each patient. The seizure onset zone (SOZ) was visually identified by the special group.

### 2.4 Concordance analysis

With the help of the open‐source software and toolboxes SPM12,^33^ Freesurfer,^30^ and 3D slicer,^32^ preoperative high‐resolution MRI images and MEG images were registered with postoperative CT or MRI images. The resection size was reconstructed using the CT images. A detailed analysis of the relationship between MEG findings and surgery and the outcome was performed by visually evaluating whether the respective MEG localizations had been resected completely, or not resected, as in previous studies.^16^, ^34^, ^35^ For single dipole cluster localizations, a tolerance of 1 cm within the border was included to define a complete resection.^35^ Spurious outlier localizations were not taken into consideration. In addition, the MEG dipoles located beyond 1 cm of the surgical resection border were defined as non‐complete resection. Since multiple dipole clusters and scattered dipoles are distributed beyond the surgical resection border, they are considered as noncomplete resection.

Concordance analysis between MEG results and other diagnostic modalities (MRI, PET, interictal EEG) were evaluated on a lobar level. Concordant levels were classified into concordance and discordance.^35^ Concordance occurred when MEG findings were included or overlapped at least one lobe in other diagnostic modalities. Discordance occurred when MEG findings did not show an overlap with other diagnostic modalities.

Spatial concordance between MEG clusters and SEEG findings was assessed at the sublobar region level, similar to previous studies.^36^ First, we analyzed whether the brain regions indicated by MEG dipoles were sampled by the SEEG electrodes, which were divided into the complete sample and partial sample. The degree of concordance between MEG and SEEG findings was then classified into complete concordance and partial concordance. If the MEG findings were included in the SOZ indicated by SEEG contacts, the findings would be regarded as complete concordance. Partial concordance occurred when MEG findings had an overlap of at least one sublobe in the SOZ but also showed differences.

### 2.5 Data analysis

Surgical outcome was evaluated by a neurologist based on the results of outpatient revisits or telephone follow‐up. Patients were classified into two groups using the modified Engel's classification: completely seizure‐free (Engel's class Ia) and not seizure‐free (Engel's class Ib–IV).^37^ The association between the surgical outcome and MEG dipole classifications, as well as MEG resection, was evaluated using Fisher's exact test. To investigate the role of MEG localizations when MRI findings are not consistent with the surgical resection extent, we performed the following analysis: discordance between MRI findings and surgical resection extent was defined as an abnormal signal on the MRI not completely located in the surgical resection area on a lobar level, or there is no abnormal signal on the MRI. In the subgroup of patients with discordant MRI findings and the extent of surgical resection (*n* = 24), we analyzed the relationship between MEG resection extent and surgical outcome using Fisher's exact test. To elaborate the role of MEG in facilitating the precise positioning of SEEG, we analyzed the surgical outcome of patients in whom the brain regions indicated by MEG dipoles were completely or partially sampled by the SEEG electrodes. We further analyzed the surgical outcome of patients whose MEG and SEEG findings were in complete or partial concordance. Fisher's exact test was used to verify whether there was a statistical difference between groups.

## 3 RESULTS

### 3.1 Characteristics of patients

Among the 854 patients reviewed, 39 (4.6%) patients were included in this study, 26 males and 13 females. The mean age of the subjects was 18.36 (± 9.02, range 5–44) years with a mean disease duration of 9.10 (± 8.78, range 0.58–41) years. More than 80% of the patients had visible lesions on MRI. The average follow‐up duration was 28.0 (± 12.8, range 12–48) months. The detailed clinical characteristics of the patients are shown in Table 1.

**TABLE 1:** Characteristics of the subjects.

Characteristics	Cases (*n* = 39)
Age, years	18.4 (9.0)
Gender, male	26 (66.7)
Disease duration, years	9.1 (8.8)
Follow‐up, months	27.5 (13.5)
MRI, positive	32 (82.1)
Aetiologies and lesions	
FCD	14 (35.9)
Ulegyria	8 (20.5)
Tumor	4 (10.3)
Gray matter heterotopia	2 (5.1)
MCD	3 (7.7)
Negative	8 (20.5)
Outcome	
Seizure‐free	21 (53.8)
Not seizure‐free	18 (46.2)

*Note*: Values are presented as mean (SD) or number (percentage). Seizure‐free: Engle I; Not seizure‐free: Engle II–IV.

Abbreviations: FCD, focal cortical dysplasia; MCD, malformations of cortical development.

Postoperative pathological examinations showed that 14 (35.9%) patients had focal cortical dysplasia (FCD) and 9 (23.1%) had ulegyria (Table 1). The last available surgical outcome was Engel I in 21 (53.8%) patients, Engel II in 5 (12.8%), Engel III in 9 (23.1), and Engel IV in 4 (10.3%) patients (Table 1). Details of demographic and clinical profiles of the study subjects are shown in Table S1.

### 3.2 MEG localizations

Among the 39 cases, a single cluster was identified in 24 cases (61.5%), multiple clusters in nine cases (23.1%), and scattered dipoles occurred in six cases (15.4%).

**FIGURE 1:**
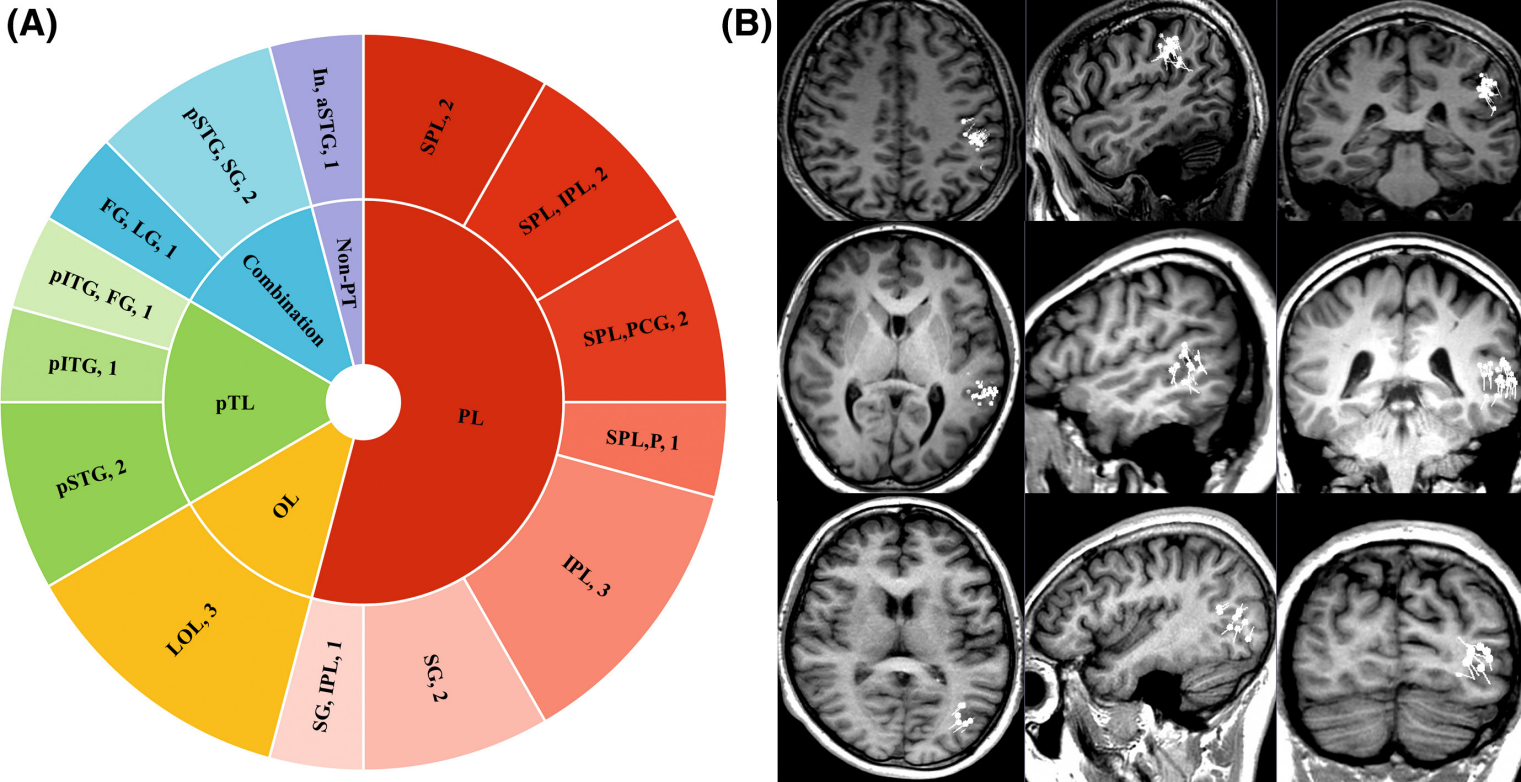
Localizations of single MEG dipole cluster in cerebral cortex and examples of single dipole cluster. (A) Distribution of single MEG dipole cluster in cerebral cortex. (B) The MEG images of three representative patients were selected from 24 patients with a single dipole cluster, and their dipoles were located in the parietal, posterior temporal, and occipital lobes. aSTG, anterior superior temporal gyrus; FG, fusiform gyrus; In, Insula; IPL, inferior parietal lobule; LG, lingual gyrus; LOL, Lateral occipital lobe; non‐PT, nonposterior cortex; OL, Occipital Lobe; P, precuneus; PCG, posterior central gyrus; pITG, posterior inferior temporal gyrus; PL, parietal lobe; pSTG, posterior superior temporal gyrus; pTL, posterior temporal lobe; SG, supramarginal gyrus; SPL, superior parietal lobule.

Among the cases with a single dipole cluster, MEG localizations included the parietal lobe in 13 cases, the occipital lobe in three cases, the posterior temporal lobe in four cases, and a combination in three cases. Details of the localizations of the single MEG dipole clusters in the cerebral cortex are shown in Figure 1.

### 3.3 MEG and epilepsy surgery

Among patients with a single cluster in MEG, the cluster was completely resected in 18 patients, 15 of whom (83%) became seizure‐free. Noncomplete resection occurred in six patients, one of whom (17%) became seizure‐free. In addition, among the patients with multiple clusters, all nine patients were noncomplete resection cases, three of whom (33%) became seizure‐free. As for patients with scattered dipoles, all six patients were noncomplete resection cases, two of whom (33%) became seizure‐free (Table 2).

**TABLE 2:** The association of dipole classifications in MEG, resection, and surgical outcome.

	Single cluster	Multiple cluster	Scatter	Total
Complete MEG resection				
Seizure‐free	15	—	—	15
Not seizure‐free	3	—	—	3
Not complete MEG resection				
Seizure‐free	1	3	2	6
Not seizure‐free	5	6	4	15

**FIGURE 2:**
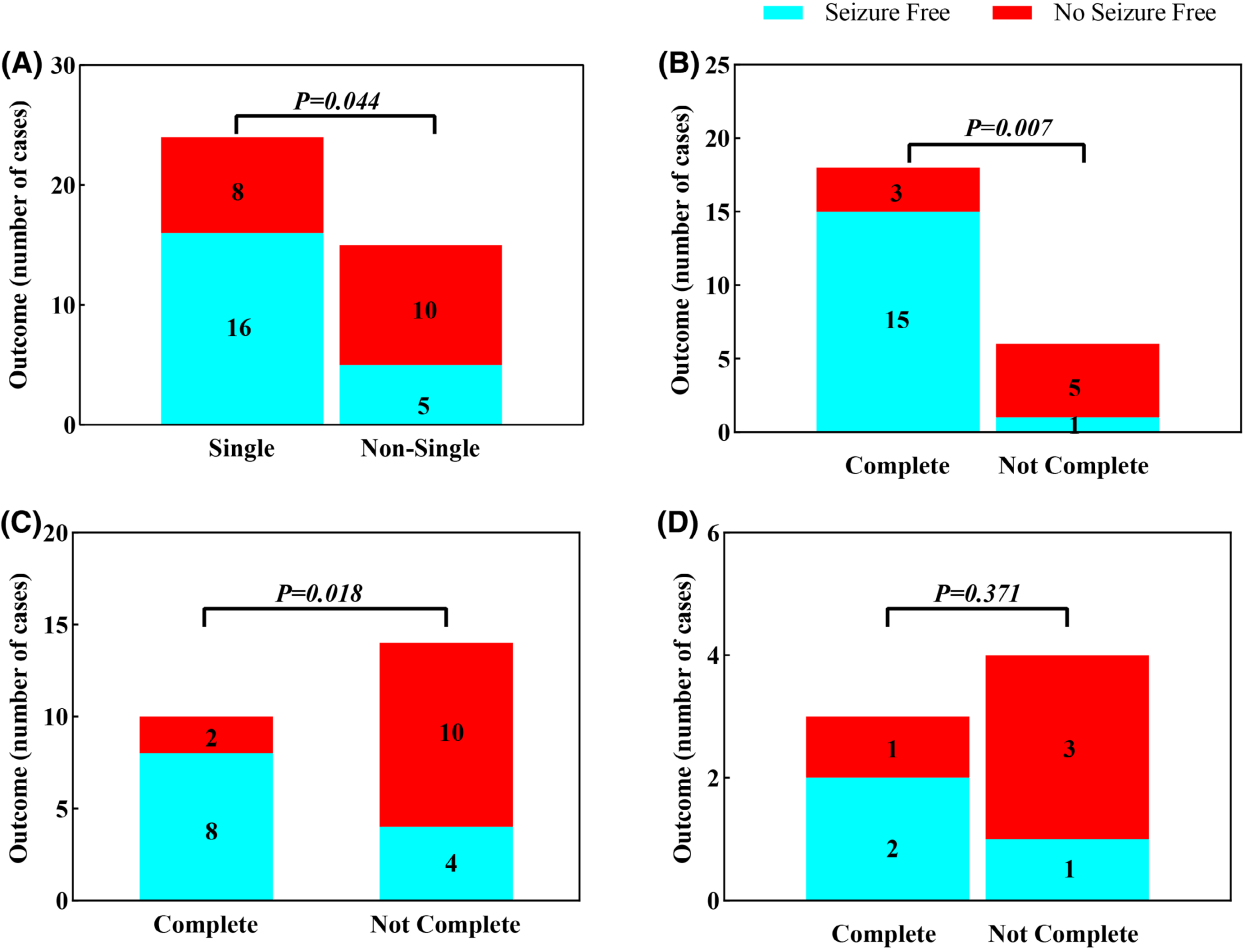
The association of single dipole cluster, MEG resection, and surgery outcome. (A) comparison of outcome between patients with and without single dipole cluster in MEG; (B) in patients with MEG single dipole cluster, comparison of outcome between patients with complete MEG dipole cluster resection or not; (C) among patients with discordant findings between MRI and surgical resection, the association of MEG resection and outcomes; (D) among patients with negative MRI findings, the association of MEG resection and outcome. Complete, complete resection of MEG dipoles; Not complete, not complete resection of MEG dipoles; Not single, including multiple MEG dipole clusters and scattered dipoles; Single, single MEG dipole cluster.

**FIGURE 3:**
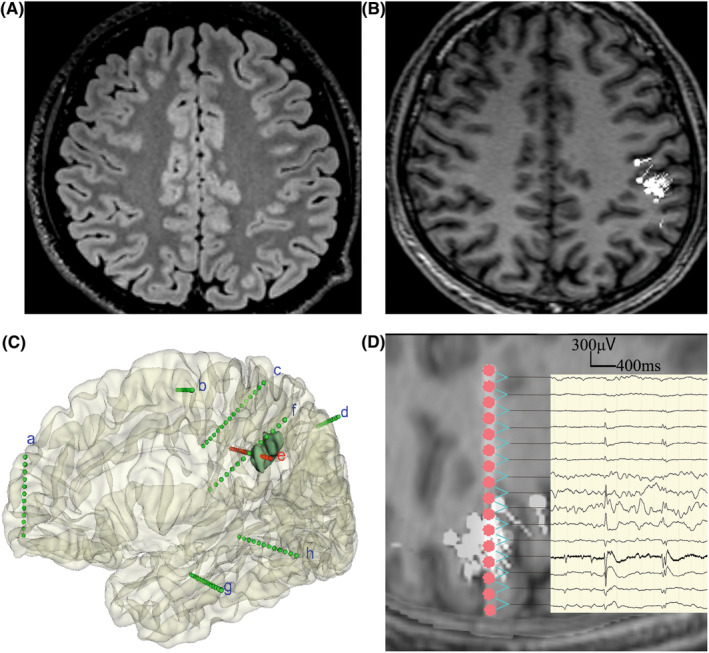
MEG navigate resection in a patient with negative MRI findings. A male patient, 22 years old, with a 7‐year history of epilepsy. This patient had a complete resection of MEG dipole cluster. At the 12‐month follow‐up visit, this patient achieved a favorable clinical outcome (Engel Class I). (A) The result of MRI in fluid‐attenuated inversion recovery sequences; (B) the results of MEG: a single cluster in the supramarginal gyrus; (C) 3D reconstructions of participants' brains and SEEG electrode contacts, the green part is the resection lesion, the electrode contacts are shown by pink or greed points; (D) a combination of the MEG‐electrode‐SEEG wave, the electrode contacts are shown by pink points, while the intracranial EEG waveforms are shown in bipolar montages beside the contacts, in one‐to‐one correspondence, the MEGSSs are shown in white points with small tails.

The association between surgery outcome and MEG dipole classifications, as well as MEG resection, was evaluated using the chi‐squared test or Fisher's exact test. In total, 66.7% of patients with single dipole clusters became seizure‐free, which was significantly higher than patients without single clusters (33.3%) (*p* = 0.044) (Figure 2A). There was no statistical significance when MEG dipole clusters were divided into three groups. Further analysis of the MEG resection and surgery outcome among patients with single dipole clusters found that complete MEG resection (83.3%) had a more favorable surgical outcome than noncomplete resection (16.7%) (*p* = 0.007) (Figure 2B). Figure 3 shows an example case with a single dipole cluster and complete resection who achieved a favorable surgical outcome at the 17‐month follow‐up visit (Engel I).

### 3.4 Concordance between MEG and diagnostic modalities versus outcome

MEG findings were concordant with MRI findings in 24 of the 39 cases (61.5%), 16 (66.7%) of whom became seizure‐free. Five of 15 (33.3%) patients with discordant MEG and MRI findings became seizure‐free. Cases with concordant MRI and MEG findings achieved a significantly more favorable surgical outcome (*p* = 0.044). No significant relationship was found between surgical outcome and concordant levels when combining MEG and any other diagnostic modalities (PET and interictal EEG) (Table 3). We further analyzed the surgical outcome when MEG findings were concordant with more than one diagnostic modality. MEG findings were concordant with both MRI and PET findings in 12 of 30 cases (40.0%), 9 (75.0%) of whom became seizure‐free. MEG findings were discordant with MRI or PET in 18 of 30 cases (60.0%), 6 (33.3%) of whom became seizure‐free. Cases with MEG concordant with both MRI and PET findings achieved a better surgical outcome (*p* = 0.030). Furthermore, a more favorable surgical outcome was not found between MEG findings and other combinations of diagnostic modalities (Table 3).

**TABLE 3:** Concordance between MEG and diagnostic modalities versus surgical outcome.

	Total	Seizure‐free	Not seizure‐free	*p*
MEG + MRI				
Concordance	24	16	8	**0.044**
Discordance	15	5	10	
MEG + PET				
Concordance	21	13	8	0.054
Discordance	9	2	7	
MEG + interictal EEG				
Concordance	32	17	15	0.591
Discordance	7	4	3	
MEG + MRI and interictal EEG				
Concordance	21	14	7	0.079
Discordance	18	7	11	
MEG + MRI and PET				
Concordance	12	9	3	**0.030**
Discordance	18	6	12	
MEG + interictal EEG and PET				
Concordance	16	9	7	0.358
Discordance	14	6	8	
MEG + interictal EEG, MRI, and PET				
Concordance	10	7	3	0.123
Discordance	20	8	12	
MEG + SEEG electrodes				
Complete sample	7	4	3	0.070
Partial sample	6	0	6	
MEG + SOZ				
Complete concordance	5	3	2	0.217
Partial concordance	8	1	7	

*Note*: Concordance: MEG findings are included or overlapped at least one lobe in the diagnostic modalities; Discordance: MEG findings do not show an overlap with the diagnostic modalities. Complete sample: brain regions indicated by MEG dipoles were completely sampled by electrodes of SEEG; partial sample: brain regions indicated by MEG dipoles were partially sampled by electrodes of SEEG; Complete concordance: MEG findings were included in the SOZ indicated by SEEG contacts; Partial concordance: MEG findings had an overlap of at least one lobe in SOZ, but also showed differences.

Abbreviations: EEG, electroencephalography; MEG, Magnetoencephalography; MRI, magnetic resonance imaging; PET, positron emission tomography; SEEG, stereo‐electroencephalography; SOZ, seizure onset zone identified by SEEG.

The bold values indicate that the *p*‐values are statistically significant, where *p* < 0.05.

We performed the same analysis in a subgroup of patients with a single dipole cluster (*n* = 24); MEG findings were concordant with MRI findings in 16 of 24 cases (66.7%), 14 (87.5%) of whom became seizure‐free. Discordant MEG and MRI findings occurred in 8 of 24 cases (33.3%), 2 (25.0%) of whom became seizure‐free. Cases with concordant MRI and MEG findings achieved a significantly more favorable surgical outcome (*p* = 0.005). No significant relationship was found between surgical outcome and concordant levels when combining MEG and any other diagnostic modalities (PET and interictal EEG) (Table S2). MEG findings were concordant with both MRI and interictal EEG findings in 15 of 24 cases (62.5%), 13 (86.7%) of whom became seizure‐free. MEG findings were discordant with MRI or interictal EEG findings in 9 of 24 cases (37.5%), 3 (33.3%) of whom became seizure‐free. Patients with MEG concordant with both MRI and interictal EEG findings achieved a better surgical outcome (*p* = 0.012). However, a better outcome was not found between MEG findings and other combinations of diagnostic modalities (Table S2).

To identify the role of MEG localization when MRI findings and surgical resection extent were discordant, we further analyzed the correlation of MEG resection and surgical outcome in this subgroup of patients (*n* = 24). Patients with complete MEG resection achieved a better surgical outcome compared with patients with noncomplete MEG resection (8/10 80% vs. 4/14 28.6%, *p* = 0.018) (Figure 2C). No relationship was found between MEG resection extent and surgical outcome in patients with single dipole clusters or negative MRI findings (Figure 2D).

### 3.5 Concordance between MEG and SEEG findings versus outcome

Brain regions indicated by MEG clusters were completely sampled by the electrodes of SEEG in seven patients, four of whom became seizure‐free. In comparison, in six patients with partial sampling, none became seizure‐free (Table 3). Among patients where the MEG findings were in complete concordance with the SOZ indicated by SEEG, three out of five patients became seizure‐free. In contrast, when the two findings were only partially concordant, seizure‐free status was achieved in one out of eight patients (Table 3). There was no statistical difference between the above two analysis methods.

## 4 DISCUSSION

In this study, we assessed 39 patients with PCE who underwent surgical treatment to investigate the role of MEG in preoperative localization and postoperative outcome prediction in patients with PCE.

A notable discovery in our research is that different MEG dipole clusters have varying implications for the surgical outcome of patients with PCE. Specifically, when MEG showed a single cluster, the likelihood of being postoperatively seizure‐free was significantly increased. Previous studies have also associated a single cluster in MEG with a favorable prognosis following epilepsy surgery.^29^, ^35^ Our study specifically addressed the role of a single cluster in the prognosis of epilepsy surgery among patients with PCE, which is a subtype of epilepsy known for its challenges in localizing the EZs A previous study reported on the relationship between MEG and SEEG's interictal spikes in patients with PCE and found that in cases of focal epilepsy, MEG provided a good evaluation of the interictal spikes.^38^ To the best of our knowledge, our study is the first to demonstrate a prominent correlation between a single dipole cluster in MEG and favorable surgical outcomes in patients with PCE. Furthermore, we conducted an analysis of the correlation between the extent of MEG resection and epilepsy surgical outcomes in a subgroup of patients with a single dipole cluster. We found that complete resection of the single cluster was significantly associated with being seizure‐free in patients with PCE (Engel Ia).

The presence of dipole clusters can offer insights into the underlying pathology and serve as a guide for subsequent surgical planning. Previous studies have shown that patients with a single dipole cluster^34^, ^39^, ^40^, ^41^ and those with dipoles confined to the same lobe^42^ tend to have more favorable postoperative outcomes. Moreover, a single cluster is more likely to overlap with the SOZ, while multiple clusters may indicate a widespread epileptic network.^39^ These findings are supported by recent research that linked single clusters with better operative outcomes.^29^ A very recent study in MRI‐negative pediatric patients, using an inter‐dipole distance of 15 mm to define “clusterness,” also showed that dipoles that clustered were closer to the SOZ (16.2 mm) than those that were scattered (30.4 mm).^43^

Concordant presurgical findings are predictors of good postsurgical outcomes.^44^ Therefore, we evaluated the overlap of MEG and other presurgical diagnostics (MRI, PET, and interictal EEG). Patients with concordant MRI and MEG findings achieved a significantly favorable surgical outcome compared to patients without concordant MRI and MEG findings (66.7% vs. 33.3%, *p* = 0.044). In the subgroup of patients with a single dipole cluster, this difference becomes even more striking (87.5% vs. 25.0%, *p* = 0.005). A prospective study of patients with FCD II by Kasper et al.^45^ suggested the high value of conducting a combined MEG–MRI approach in the presurgical workup and the resection strategy in patients with FCD II‐related epilepsy. Our study demonstrated the same conclusions in patients with PCE.

In patients in which MEG and the presurgical evaluation were not completely concordant, MEG may suggest the involvement of additional areas, which were not indicated by other methods.^35^ This interpretation is supported by studies comparing MEG and invasive EEG.^29^, ^46^, ^47^ It is widely known that MRI is an important preoperative evaluation tool; however, MRI findings may be negative or different from other preoperative evaluation methods, making it difficult to localize the epileptic lesion. Therefore, in cases with discordant MRI findings and surgical resection extent, our research showed that complete resection of the MEG‐positive brain region achieved a better surgical outcome in PCEs. Our previous study also showed that MEG was a useful clinical tool for the preoperative evaluation of MRI‐negative operculo‐insular epilepsies.^39^ In addition, a previous study showed that MEG was a useful supplement for patients with MRI‐negative epilepsy.^28^, ^48^, ^49^, ^50^

Intracranial recordings, such as SEEG, are regarded as the gold standard to delineate the epileptogenic zone for surgical resection^51^ in PCE. The current study showed that complete concordance of MEG dipoles with SOZ indicated by SEEG was associated with a better surgery outcome, as in some previous studies.^36^, ^52^ However, partial sampling of MEG‐positive regions by SEEG electrodes was associated with a worse surgical outcome. For example, in some patients of our study, electrodes were implanted only unilaterally according to diagnostic modalities that indicated unilateral abnormalities in the preoperative evaluations, while the MEG indicated bilateral dipole clusters; these patients failed in becoming seizure‐free. We speculate that the poor prognosis may be due to the failure of electrodes implanted into additional brain regions suggested by MEG.^14^ In addition, the epileptic brain network may be more complex when MEG findings are not concentrated or different from other diagnostic modalities. Therefore, our results indirectly suggested that MEG diploes could avoid the locations of spikes missed by SEEG, which would improve the presurgical evaluation of the epileptogenic zone. It was a pity that the above results in this study were not statistically significant, which may be due to the small amount of data.

Several limitations to this study should be acknowledged. MEG analysis was conducted retrospectively, which entails the limitations of a retrospective study design. Moreover, patient selection was not standardized, potentially introducing bias. It is also important to consider that the type of pathology could influence surgical outcomes,^53^ although this aspect was not extensively explored due to the small sample size. This study did not distinguish the dipole direction‐related problems. It is generally believed that the direction of the dipole indicates the direction of the current, which should be perpendicular to the sulcus gyrus.^5^ However, since the gyrus does not grow in a straight line, different dipole directions might be related to the curved nature of the sulcus gyrus itself. Furthermore, our study did not include epilepsy patients associated with benign MEG‐unique variants. For example, benign MEG‐unique variants can be observed in the posterior temporal region over the perisylvian area, and dipoles localized here are typically benign, especially if they are bilateral or have 180 degrees opposing orientations.^54^ This type of epilepsy was not involved in our study because the orientation of the single dipole cluster in our study was always consistent. In addition, there are limitations inherent in the present MEG technology, such as the special recording environment, strict restriction of subject movement, and high maintenance costs. It is necessary to develop more sophisticated techniques to enhance the efficacy of MEG for electrode implantation, as well as the potential impact of these techniques on the resulting outcomes. The wearable MEG devices, such as MEG based on optically pumped magnetometers (OPMs),^55^ may be able to change the landscape of epilepsy.

## 5 CONCLUSION

Our research highlights that MEG can provide additional valuable information in surgical candidate selection, epileptogenic zone localization, the electrode implantation schedule, and final surgical planning in patients with PCE. Single dipole clusters in MEG and concordant findings between MEG and MRI could predict a better surgical outcome, especially when MEG dipole clusters were completely resected.

## AUTHOR CONTRIBUTIONS

Guiliang Hao and Tao Yu contributed to the conception and design of the study. Xueyuan Wang, Runshi Gao, Yansong Xue, Xiating Zhang, Duanyu Ni, Wei Shu, Liang Qiao, and Liu He contributed to the acquisition and analysis of data. Guiliang Hao contributed to the drafting of the text and preparing the figures.

## FUNDING INFORMATION

This study was funded by the STI2030‐Major Projects (2021ZD0201605) and the National Natural Science Foundation of China (82271494).

## CONFLICT OF INTEREST STATEMENT

The authors declare no conflicts of interest.

## PATIENT CONSENT STATEMENT

All patients provided written informed consent, and legal guardians provided consent for underage subjects.

## Supporting information


Tables S1–S2

## Data Availability

Anonymized data generated during the current study are available from the corresponding author upon reasonable request.
