# Supplementary material for: The role of magnetoencephalography in preoperative localization and postoperative outcome prediction in patients with posterior cortical epilepsy
Source: CNS Neurosci Ther. 2024 Feb 8;30(2):e14602. doi: 10.1111/cns.14602 (PMC10853654; doi:10.1111/cns.14602)
Supplement: Supplementary file 1 — Tables S1–S2 [file CNS-30-e14602-s001.docx]

**Supplementary table 1.** Demographic and clinical profiles of study subjects

| Patient | Sex/age,years | History, years | Auras or initial semiology | MEG | | MRI | PET/CT | Interictal EEG | Surgical resection | Surgical outcome, Engel | Pathology |
| --- | --- | --- | --- | --- | --- | --- | --- | --- | --- | --- | --- |
|  |  |  |  | Dipoles classifications | Dipoles locations |  |  |  |  |  |  |
| 1 | M/5 | 3.5 | Epigastric sensation; nausea; fear | Multiple cluster | B-frontal and parietal | N | - | L-frontal and temporal | L-parietal | II | Negative |
| 2 | F/20 | 5 | Focal unaware  seizure | Single cluster | L-posterior superior temporal and supramarginal | L-parietal and occipital | - | L-temporal | L-posterior temporal and parietal | I | Ulegyria |
| 3 | M/24 | 20 | Dizziness | Single cluster | L-supramarginal | R-parietal; B-occipital | B-frontal, temporal; L-parietal | R-temporal and parietal | L-parietal | I | FCD Ib |
| 4 | F/11 | 8 | Headache; fear | Multiple cluster | B-temporal | R-frontal | R-temporal and occipital | R-frontal | R-posterior temporal and occipital | II | MCD |
| 5 | M/12 | 5 | Atonic seizure | Scatter | B-occipital | L-occipital | L-frontal and parietal | B-posterior temporal, parietal and occipital | L-posterior temporal and occipital | II | Negative |
| 6 | M/31 | 8 | Palpitate; nausea; photophobia | Scatter | L-parietal and occipital | L-occipital | L-temporal | L-posterior temporal, parietal and occipital | L-occipital | IV | Negative |
| **7** | F/7 | 7 | GTCS | Single cluster | L-superior parietal | L-temporal and parietal | L-temporal and parietal | L-posterior temporal, parietal and occipital | L-posterior temporal, parietal and occipital | III | MCD |
| 8 | M/25 | 13 | Automatisms | Single cluster | L-posterior superior temporal | L-temporal | R-frontal and temporal | L- posterior temporal | L-posterior temporal | I | FCD IIa |
| 9 | M/29 | 20 | Palpitate; blurring | Scatter | B-temporal | L-temporal, parietal and occipital | L-temporal and parietal | L-frontal | L-parietal and occipital | I | heterotopia |
| 10 | M/14 | 1 | Focal unaware  seizure; soliloquize; | Single cluster | R-supramarginal gyrus and inferior parietal | R-parietal | - | B-frontal and parietal; L-temporal | R-parietal | I | Tumor |
| 11 | M/23 | 7 | Chest tightness; blink | Scatter | B-temporal | L-parietal | - | L-temporal and parietal | L-occipital | I | FCD Ia |
| 12 | M/33 | 16 | Palpitate; visual illusion | Single cluster | R-inferior parietal | N | R-temporal, parietal and occipital | R-hemisphere | R-posterior temporal and parietal | II | Negative |
| 13 | M/28 | 5 | L-hand numbness | Single cluster | R-superior and inferior parietal | R-parietal and occipital | R-temporal | R-temporal, parietal and occipital | R-parietal | I | FCD IIb |
| 14 | M/13 | 3 | Visual illusion; palpitate; nausea; Déjà vu | Single cluster | R-inferior parietal | R-temporal and parietal | R-temporal and parietal | R- posterior temporal, parietal and occipital | R-posterior temporal, parietal and occipital | I | Negative |
| 15 | M/10 | 8 | Epigastric sensation | Single cluster | R-posterior inferior temporal | N | R-temporal and occipital | R- posterior temporal and occipital | R-posterior temporal, parietal and occipital | II | Ulegyria |
| 16 | F/29 | 5 | Focal unaware  seizure; automatisms | Single cluster | R-posterior superior temporal | R-parietal | R-parietal | R-temporal | R-posterior temporal and parietal | III | Negative |
| 17 | F/13 | 3 | soliloquize | Single cluster | L-insula and superior temporal | L-temporal, parietal and occipital | L-temporal, parietal and occipital | L-temporal | L-posterior temporal and occipital | I | Negative |
| 18 | M/18 | 4 | Visual illusion | Multiple cluster | B-frontal and insula | R-parietal | B-frontal and parietal | B-occipital | R-parietal | I | Tumor |
| 19 | M/18 | 18 | Somatosensory aura | Scatter | R-parietal | L-temporal and parietal | L-temporal and parietal | L-frontal, temporal and parietal | R-parietal | III | FCD III d |
| 20 | M/22 | 5 | L-arm automatisms | Single cluster | L-supramarginal | N | B-parietal; L-temporal | Little abnormal activities | L-parietal | I | FCD IIa |
| 21 | M/17 | 9 | Visual illusion | Single cluster | R-superior parietal, Inferior parietal and precuneus | R-temporal and occipital | R-temporal; L-occipital | R-temporal | R-occipital | IV | FCD Ia |
| 22 | M/13 | 5 | Blurring; visual illusion | Scatter | R-temporal and occipital | N | R-temporal | R-posterior temporal and occipital | R-posterior temporal and occipital | IV | FCD Ia |
| 23 | F/16 | 13 | Focal unaware  seizure | Single cluster | L-posterior superior temporal and supramarginal | L-temporal and parietal | - | L-temporal | L-parietal | I | Tumor |
| 24 | M/17 | 4 | Focal unaware  seizure | Single cluster | L-inferior parietal | L-parietal | L-parietal and occipital | L-parietal and occipital | L-parietal and occipital | I | Ulegyria |
| 25 | M/11 | 4 | Tonic | Multiple cluster | B-temporal and parietal | R-frontal | L-frontal | R-hemisphere | R-posterior temporal and parietal | IV | FCD Ia |
| 26 | F/20 | 3 | Visual illusion | Single cluster | L-posterior Inferior temporal, parahippocampal and fusiform | L-temporal | L-temporal and parietal | L-posterior temporal and occipital | L-posterior temporal and occipital | III | Negative |
| 27 | M/9 | 8 | L-limbs myoclonic | Single cluster | L-superior parietal and postcentral gyrus | L-frontal | L-temporal and parietal | B-parietal | L-parietal | III | Ulegyria |
| 28 | M/9 | 2 | Blurring; visual illusion | Single cluster | R-lateral occipital | R-occipital | R-temporal and occipital | R-posterior temporal, parietal and occipital | R-posterior temporal and occipital | I | Ulegyria |
| 29 | F/22 | 9 | Focal unaware  seizure | Single cluster | L-superior and inferior parietal | L-frontal, temporal and parietal | L-temporal and parietal | L-posterior temporal and occipital | L-parietal | I | Ulegyria |
| 30 | M/7 | 0.83 | Automatisms | Single cluster | R-superior parietal and postcentral gyrus | R-parietal | - | R-temporal and parietal | R-parietal | I | FCD IIId |
| 31 | F/11 | 2 | Visual illusion | Single cluster | R-fusiform gyrus and lingual gyrus | B-temporal | R-temporal and occipital | B-posterior temporal and occipital | R-posterior temporal and occipital | I | FCD Ia |
| 32 | M/5 | 0.58 | Focal unaware  seizure | Single cluster | R-lateral occipital | R-parietal and occipital | - | R-posterior temporal, parietal and occipital | R-posterior temporal, parietal and occipital | I | Ulegyria |
| 33 | F/15 | 2 | Focal unaware  seizure | Multiple cluster | R-parietal and temporal | R-parietal | R-parietal and temporal | R-frontal, parietal and temporal | R-parietal and occipital | I | Tumor |
| 34 | M/31 | 30 | L-hand clonic | Multiple cluster | B-temporal and parietal | L-frontal, temporal and occipital | - | L-parietal | L-posterior temporal and parietal | III | FCD IIb |
| 35 | M/16 | 11 | L-hand numbness | Multiple cluster | L-temporal and parietal | B-frontal, parietal and occipital | - | L-frontal, parietal and temporal | L-posterior temporal and parietal | III | FCD Ia |
| 36 | F/34 | 26 | Blurring; dizziness; nausea | Multiple cluster | R-temporal and frontal | N | R-temporal, parietal and occipital | R-posterior temporal, parietal and occipital | R-parietal and occipital | I | FCD Ia |
| 37 | F/44 | 41 | Focal unaware  seizure; left-hand automatisms | Multiple cluster | L-temporal and occipital | L-temporal, parietal and occipital | L-temporal, parietal and occipital | L-posterior temporal, parietal and occipital | L-posterior temporal, parietal and occipital | III | MCD |
| 38 | M/15 | 3 | Blurring | Single cluster | L-lateral occipital | N | L-temporal and occipital | L-occipital | L- posterior temporal and occipital | I | heterotopia |
| 39 | F/19 | 17 | L-hand numbness | Single cluster | L-superior parietal | R-temporal | R-temporal and parietal | B-frontal, temporal and parietal | R-parietal and occipital | III | Ulegyria |

Abbreviations: F, female; M, male; MEG, Magnetoencephalography; PET/CT, positron emission tomography/computerized tomography; GTCS, generalized tonic-clonic seizures; B, bilateral; L, left; R, right; N, negative; MCD, malformations of cortical development; FCD, focal cortical dysplasia.

Supplementary table 2: Concordance between MEG and diagnostic modalities versus surgical outcome among patients with single dipole cluster

|  | **Total** | **Seizure-free** | **Not seizure-free** | ***P*** |
| --- | --- | --- | --- | --- |
| MEG+MRI |  |  |  |  |
| Concordance | 16 | 14 | 2 | **0.005** |
| Discordance | 8 | 2 | 6 |  |
| MEG+PET |  |  |  |  |
| Concordance | 14 | 9 | 5 | 0.336 |
| Discordance | 5 | 2 | 3 |  |
| MEG+ interictal EEG |  |  |  |  |
| Concordance | 21 | 14 | 7 | 1.000 |
| Discordance | 3 | 2 | 1 |  |
| MEG+MRI and interictal EEG |  |  |  |  |
| Concordance | 15 | 13 | 2 | **0.012** |
| Discordance | 9 | 3 | 6 |  |
| MEG+MRI and PET |  |  |  |  |
| Concordance | 9 | 7 | 2 | 0.115 |
| Discordance | 10 | 4 | 6 |  |
| MEG+ interictal EEG and PET |  |  |  |  |
| Concordance | 12 | 7 | 5 | 0.663 |
| Discordance | 7 | 4 | 3 |  |
| MEG+ interictal EEG, MRI, and PET |  |  |  |  |
| Concordance | 8 | 6 | 2 | 0.208 |
| Discordance | 11 | 5 | 6 |  |

Concordance: MEG findings are included or overlapped at least one lobe in the diagnostic modalities; Discordance: MEG findings do not show an overlap with the diagnostic modalities.
